# A Mobile Social Networking App for Weight Management and Physical Activity Promotion: Results From an Experimental Mixed Methods Study

**DOI:** 10.2196/19991

**Published:** 2020-12-08

**Authors:** Liliana Laranjo, Juan C Quiroz, Huong Ly Tong, Maria Arevalo Bazalar, Enrico Coiera

**Affiliations:** 1 Australian Institute of Health Innovation Macquarie University Sydney Australia; 2 Westmead Applied Research Centre Faculty of Medicine and Health The University of Sydney Sydney Australia; 3 Centre for Big Data Research in Health University of New South Wales Sydney Australia

**Keywords:** mobile apps, fitness trackers, exercise, social networking, body weight maintenance, mobile phone

## Abstract

**Background:**

Smartphone apps, fitness trackers, and online social networks have shown promise in weight management and physical activity interventions. However, there are knowledge gaps in identifying the most effective and engaging interventions and intervention features preferred by their users.

**Objective:**

This 6-month pilot study on a social networking mobile app connected to wireless weight and activity tracking devices has 2 main aims: to evaluate changes in BMI, weight, and physical activity levels in users from different BMI categories and to assess user perspectives on the intervention, particularly on social comparison and automated self-monitoring and feedback features.

**Methods:**

This was a mixed methods study involving a one-arm, pre-post quasi-experimental pilot with postintervention interviews and focus groups. Healthy young adults used a social networking mobile app intervention integrated with wireless tracking devices (a weight scale and a physical activity tracker) for 6 months. Quantitative results were analyzed separately for 2 groups—underweight-normal and overweight-obese BMI—using *t* tests and Wilcoxon sum rank, Wilcoxon signed rank, and chi-square tests. Weekly BMI change in participants was explored using linear mixed effects analysis. Interviews and focus groups were analyzed inductively using thematic analysis.

**Results:**

In total, 55 participants were recruited (mean age of 23.6, SD 4.6 years; 28 women) and 45 returned for the final session (n=45, 82% retention rate). There were no differences in BMI from baseline to postintervention (6 months) and between the 2 BMI groups. However, at 4 weeks, participants’ BMI decreased by 0.34 kg/m^2^ (*P*<.001), with a loss of 0.86 kg/m^2^ in the overweight-obese group (*P*=.01). Participants in the overweight-obese group used the app significantly less compared with individuals in the underweight-normal BMI group, as they mentioned negative feelings and demotivation from social comparison, particularly from upward comparison with fitter people. Participants in the underweight-normal BMI group were avid users of the app’s self-monitoring and feedback (*P*=.02) and social (*P*=.04) features compared with those in the overweight-obese group, and they significantly increased their daily step count over the 6-month study duration by an average of 2292 steps (95% CI 898-3370; *P*<.001). Most participants mentioned a desire for a more personalized intervention.

**Conclusions:**

This study shows the effects of different interventions on participants from higher and lower BMI groups and different perspectives regarding the intervention, particularly with respect to its social features. Participants in the overweight-obese group did not sustain a short-term decrease in their BMI and mentioned negative emotions from app use, while participants in the underweight-normal BMI group used the app more frequently and significantly increased their daily step count. These differences highlight the importance of intervention personalization. Future research should explore the role of personalized features to help overcome personal barriers and better match individual preferences and needs.

## Introduction

### Background

Obesity and physical inactivity are major societal challenges and significant contributors to the global burden of disease and health care costs [1-3]. Globally, excess body weight and obesity account for approximately 4 million deaths in a year and 120 million disability-adjusted life years [1]. Effective weight management interventions usually involve a combination of behavioral strategies focusing on dietary changes and physical activity [4]. Notably, physical activity is also independently associated with better health outcomes in a dose-response manner [5], however, more than one-fourth of adults globally are insufficiently active [6]. Promoting physical activity and weight management (ie, preventing weight gain to unhealthy levels and promoting weight loss in individuals with excess weight) are important behavioral strategies for better health outcomes.

Addressing obesity and physical inactivity requires a combination of environmental approaches and effective behavior change interventions that can be easily disseminated. Apps and fitness trackers are becoming pervasive in the daily lives of people, with smartphone ownership surpassing three-fourth of the population and activity trackers being used by one-third of adults in the United States and United Kingdom [7,8]. These mobile technologies enable the automation of effective behavior change techniques—weight and physical activity self-monitoring and feedback [9-12]—showing potential in weight management and physical activity interventions [13,14].

Although mobile technologies can facilitate self-monitoring and feedback, behavior change is also heavily regulated by social processes. Online social networks—platforms that allow individuals to create their own personal profile and build a network of connections with other users—can facilitate behavior change [15-18] and have shown potential in weight management and physical activity interventions [19-22]. Online social networks enable the delivery of several social functions, including support and social comparison, which have been associated with increased physical activity [23-25] and greater weight loss in weight management interventions [26,27]. However, user preferences regarding social features seem to be mixed: for some users, these features promote engagement with the intervention, whereas for other users, they are less enjoyable or even disliked [20,28]. Key questions remain as to which intervention features and social network characteristics are more effective and engaging for users in weight management and physical activity promotion [15,18].

### Aims

This 6-month pilot study of a social networking mobile app connected to wireless weight and activity tracking devices has 2 main aims: (1) to evaluate changes in BMI, weight, and physical activity levels in users from different BMI categories and (2) to assess user perspectives on the intervention, particularly its online social networking component and automated self-monitoring and feedback features.

## Methods

### Study Design

This was a mixed methods study involving a pre-post quasi-experimental pilot with one arm, where participants used a social networking mobile app intervention integrated with wireless tracking devices (a weight scale and a physical activity tracker) for 6 months [29]. Ethics approval was granted by the Human Research Ethics Committee for Medical Sciences of Macquarie University. Reporting follows the mobile health (mHealth) evidence reporting and assessment (mERA) checklist [30], the transparent reporting of evaluations with nonrandomized designs (TREND) statement for reporting of nonrandomized evaluations of behavioral and public health interventions [31], the COnsolidated Criteria for Reporting Qualitative Research (COREQ) checklist [32], and guidelines for good reporting of mixed methods studies [33] (Multimedia Appendices 1-3).

### Study Sample and Recruitment

Eligible study participants were consenting healthy adults who were able to speak, write, and read English, were between 19 and 35 years of age, owned a mobile phone (iOS or Android) with internet access, and had Wi-Fi connection at home. Exclusion criteria were pregnancy or breastfeeding, BMI below 17 kg/m^2^, prior history of eating disorders, and having diabetes or other comorbid conditions that could have impacted study participation.

Students and staff from Macquarie University (Sydney, Australia) were recruited via posters and flyers distributed around campus as well as via a Facebook post on the university’s page (Multimedia Appendix 4). Recruitment was completed in April 2017 and followed a purposive convenience sampling technique to test the intervention in a diverse BMI sample. To enable a comparable number of individuals in lower and higher BMI groups (at least 20 individuals in each of those 2 groups), recruitment of individuals in the normal BMI range was stopped earlier, whereas recruitment for the higher BMI groups continued. A sample size of at least 40 individuals was pragmatically chosen to enable pilot testing of the intervention based on available funding. At baseline, participants were invited to attend the initial study session at a research center on campus, where they signed the consent form and filled in a questionnaire about their demographic characteristics. A complete description of study procedures and interventions can be found in published papers [29,34,35].

### Intervention

The intervention consisted of a mobile app (fit.healthy.me) [29,34,35] designed by the research team, which was integrated with 2 wireless devices (Fitbit Aria weight scale and Fitbit Flex 2 physical activity tracker, connected via the Fitbit app). The goal of the intervention was to promote physical activity and support weight management in users of any physical activity level or BMI group. In fit.healthy.me, the participants could compare their step count and weight with other users, in table and graphical formats (Multimedia Appendix 5). Furthermore, users were able to interact and provide social support to each other through the use of messaging and a social forum, as well as *follow* particular *buddies* with whom they identified more closely.

The intervention allowed for the delivery of several behavior change techniques [11]: self-monitoring and feedback on behavior (daily number of steps), self-monitoring and feedback on weight and BMI, instructions on how to perform the behavior, social support, and social comparison. Prompts and cues (text messages and emails every 2 weeks) were used to promote engagement with the intervention. Goal setting was not incorporated in fit.healthy.me but it was a core component of the Fitbit devices (eg, the activity tracker had a predefined daily step goal of 10,000, which could be modified by users); goal setting was neither promoted nor discouraged by researchers.

### Quantitative Data Collection and Analysis

#### Weight and BMI

The primary outcome was the difference in the average BMI between 6 months and baseline. Body weight was measured with a Fitbit Aria scale to the nearest 0.1 kg in light clothing without shoes, before and after the intervention period. Height was measured using a wall-mounted stadiometer to the nearest 0.1 cm. BMI was calculated as weight (kg)/height^2^ (m^2^). Weight and BMI were also measured by participants at several time points throughout the 6 months (participants were asked to weigh themselves daily using the Fitbit Aria scale provided for research purposes, to enable the comprehensive testing of the integration between the fit.healthy.me app and the wireless scale, and early detection of potential problems).

#### Daily Step Count

The daily step count was measured using the Fitbit Flex 2. To establish a baseline average daily step count, participants did not have access to the intervention app for the first week after enrollment. Participants were considered to have a valid step count if they wore the Fitbit for at least 10 hours on any given day. Wear time was calculated by subtracting nonwear time from 24 hours; nonwear time was defined when step counts over a period of at least 60 continuous minutes were zero (allowing for counts of less than 100 for a maximum of 2 min within that period) [35].

#### Engagement Measures

Retention was defined as attendance at the 6-month final session; participants who attended the final sessions were considered *completers*. For the fit.healthy.me app, engagement was measured by number of app sessions and frequency of usage of app features (ie, the number of times participants used each feature). One app session was defined as any activity occurring in the app until the user logged off or when 10 consecutive minutes of inactivity were reached. A participant was considered to have used a social feature if they clicked on any of *My team*, *Social forum*, and *Private messages* features.

#### Data Analysis

Quantitative results were analyzed separately for 2 groups: underweight-normal BMI (18-24.99 kg/m^2^) and overweight-obese (≥25 kg/m^2^). Missing weight and step count data were imputed using the last observation carried forward (last measurement obtained from the Fitbit devices). Independent two-sample *t* tests were used for normally distributed continuous variables. For nonnormal data, the Wilcoxon sum rank test was used (Wilcoxon signed rank test for paired within-group comparisons). Chi-square tests were used for categorical data.

We performed an exploratory linear mixed effects analysis of the weekly BMI change of each participant. The dependent variable was BMI change. We used intercepts, sex, age, and weight baseline as fixed effects. As random effects, we had intercepts for subjects as well as by-subject random slopes for the effect of time. We used 6 months of data, with the origin of the time variable (week) set at week 4 (posthoc decision), for 2 main reasons: the likelihood of observing weight changes due to the intervention would be higher at 4 weeks rather than before (given that weight loss increases with intervention duration [36]) and the amount of missing data was minimal during the first month of the intervention, allowing for a more robust model [37]. *P* values were obtained by using likelihood ratio tests of the full model with the effect in question against the model without it. Data were analyzed using R version 3.5.0 and Ime4 [38]. The significance level for all statistical tests was set at *P*<.05, two tailed, and 95% CIs were calculated where applicable.

### Qualitative Data Collection and Analysis

An interview guide [34] was pilot tested before study commencement. In the final 6-month session, 2 researchers with expertise in qualitative methods conducted individual interviews and focus groups with participants to understand their perspectives on the advantages and disadvantages of the intervention, until data saturation was reached. The interviews allowed us to understand individual perspectives, and the focus groups aimed to explore group differences and similarities. Field notes were taken throughout the interviews and focus groups. Interviews and focus groups were audio-recorded and transcribed verbatim.

All data were imported and managed in Nvivo 11 (QSR International). Data were analyzed using thematic analysis, where transcripts and field notes were read to identify and code common ideas and patterns emerging from the data [39]. Through constant comparison, codes and concepts were clustered together to form subthemes, and further abstracted to originate themes, which were then reviewed and refined [40]. The integration of results was done after quantitative and qualitative analyses were conducted through embedding of the data. Integration is presented throughout the *Discussion* section.

## Results

### Sample Characteristics

A total of 55 participants were recruited, with a mean age of 23.6 (SD 4.6) years; 51% (n=28) were female (Table 1). Most (n=24, 44%) participants had a normal BMI, 27% (n=15) were overweight, 24% (n=13) were obese, and 5% (n=3) were underweight. The mean step count per day at baseline was 9937 (SD 3527). Of the 55 recruited participants, 45 returned for the final session (study completers)—the retention rate was 82%. There were no statistically significant differences in baseline characteristics between enrolled participants and study completers.

**Table 1:** Baseline characteristics of the study sample, according to BMI categories.

Variable	BMI categories (kg/m^2^)^a^				Enrolled participants (N=55)		Study completers (n=45)	
	18-18.49 (n=3)	18.5-24.99 (n=24)	25-29.99 (n=15)	≥30 (n=13)				
Age (years), mean (SD)	22.2 (3.3)	22.2 (3.6)	25.6 (5.6)	24.1 (4.8)		23.58 (4.6)		24.2 (4.7)
Female, n (%)	2 (67)	15 (63)	4 (25)	7(25)		28 (51)		22 (49)
Weight (kg), mean (SD)	54.3 (5.0)	65.6 (7.9)	84.4 (8.0)	107.1 (22.7)		78.1 (22.3)		77.8 (21.2)
BMI (kg/m^2^), mean (SD)	19.4 (1.3)	22.6 (2.3)	27.5 (1.6)	36.9 (5.5)		26.5 (6.8)		26.7 (6.5)
Steps per day^b^, mean (SD)	8203 (2824)	9619 (1720)	12,128 (3820)	8912 (3345)		9937 (3527)		9946 (3656)

^a^According to the World Health Organization, a BMI of <18.5 is classified as underweight, 18.5-24.9 is normal, 25-29.9 is preobese, and ≥30 is obese.

^b^Analysis of variance: *P*=.03.

### Quantitative Findings

#### Weight and BMI

**Figure 1:**
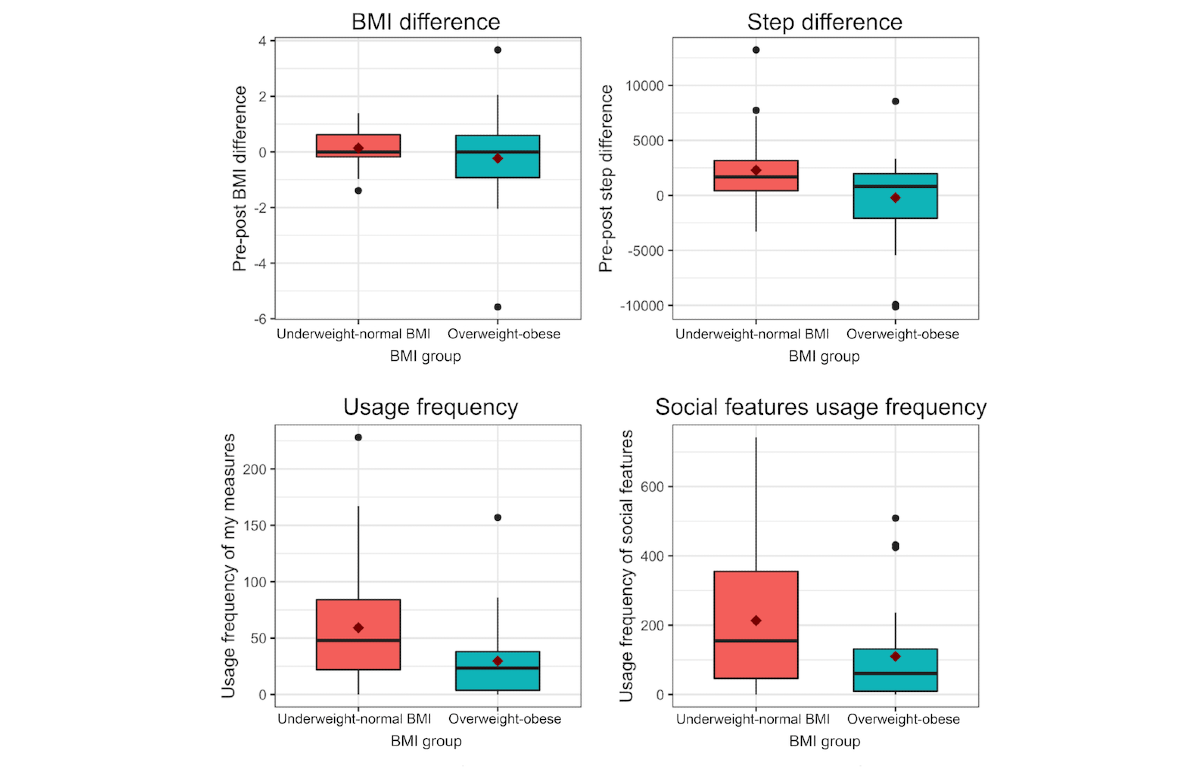
Box plots of pre-post BMI difference, pre-post step count difference, My measures use frequency, and social features use frequency.

There were no statistically significant differences in BMI from baseline to postintervention (6 months) and between the underweight-normal and overweight-obese groups (Table 2; Figure 1). Linear mixed effects analyses of the weekly BMI change of each participant are shown in Table 3. Age, pre-post step difference, and scale usage did not have an effect on BMI difference in any of the 3 groups (all participants; underweight-normal BMI; and overweight-obese; Multimedia Appendix 6). A model with sex and baseline weight as fixed effects was not statistically different from the model presented in Table 3, with just sex as a fixed effect (parameters for the model with sex and baseline weight as fixed effects are included in Multimedia Appendix 7).

At 4 weeks of the intervention, participants’ BMI decreased by 0.34 kg/m^2^ (0.86 kg/m^2^ in the overweight-obese group). Over the 6-month study, men showed a 0.32 increase in BMI relative to women during the intervention (Table 3). The variability in the rate of BMI change across all participants over the duration of the intervention was low (≤0.004), but it was 4 times higher in the overweight-obese group (BMI change variance of 0.004) compared with the underweight-normal group (BMI change variance of 0.001).

**Table 2:** Differences in characteristics of underweight-normal BMI and overweight-obese participants.

Variable		Underweight-normal BMI^a^ (n=27), mean (SD)	Overweight-obese (n=28), mean (SD)	*P* value (95% CI)^b^
Baseline steps per day		9094 (2916.2)	10,749 (3910.8)	.10 (–3299 to 255)
Pre-post step difference		2292 (3520.4)^c^	–213.7 (4023.4)^d^	*.049*^e^ (4.6 to 3891)
Pre-post BMI difference		0.13 (0.68)	–0.23 (1.58)	.30 (–0.14 to 0.82)
Scale use		111.9 (65.4)	119.4 (122)	.60 (–36 to 60)
App sessions^f^		23.5 (23.8)	15.8 (20)	.08 (–1 to 13)
**App use frequency**		1894 (1653.5)	1384.3 (1192.2)	.30 (–313 to 1100)
	My measures use	59.2 (54.8)	29.7 (35)	*.02* (3 to 45)
	Social features use^g^	213.3 (208.5)	110.1 (139)	*.04* (0 to 167)
System usability scale score^h^		60.8 (20.7)	59.4 (18.1)	.34 (–5 to 15)

^a^Using baseline measurements; normal BMI: BMI 17-24.9 kg/m^2^, overweight-obese: BMI of ≥25.

^b^Wilcoxon sum rank test.

^c^Within-group (Wilcoxon sign rank test): *P*<.001; 95% CI 898 to 3370.5.

^d^Within-group: *P=*.73; 95% CI 1365.7 to 1486.4.

^e^Italics denotes statistical significance.

^f^One app session was defined as any activity occurring in the app until the user logs off or when 10 min of inactivity are reached.

^g^Social features include *My team*, *Social forum*, *Private messages*.

^h^Only study completers (ie, participants who returned to the final session) completed the system usability scale (n=45).

**Table 3:** Linear mixed effects analysis of the weekly BMI change of each participant.

Predictors^a^	BMI											
	All participants				Underweight-normal				Overweight-obese			
	Estimate	SE	95% CI	*P* value	Estimate	SE	CI	*P* value	Estimate	SE	95% CI	*P* value
Intercept	–0.34	0.10	(–0.5 to –0.1)	*<.001* ^b^	–0.23	0.28	(–0.8 to 0.3)	.41	–0.86	0.33	(–1.5 to –0.2)	*.01*
Sex	0.32	0.14	(0.05 to 0.6)	*.02*	0.17	0.16	(–0.2 to 0.5)	.31	0.34	0.22	(–0.1 to 0.8)	.12
Weekly BMI change	–0.01	0.01	(–0.02 to 0.01)	.28	0.01	0.01	(–0.01 to 0.02)	.22	–0.02	0.01	–0.05 to 0.0)	.06

^a^Random effects: intercept variance 0.27 (all participants), 0.15 (underweight-normal), 0.34 (overweight-obese); weekly BMI change variance 0.002 (all participants), 0.001 (underweight-normal), 0.004 (overweight-obese); covariance intercept–weekly BMI change 0.37 (all participants), 0.06 (underweight-normal), 0.37 (overweight-obese).

^b^Italics denotes statistical significance.

#### Daily Step Count

The underweight-normal group increased their daily step count over the 6-month study duration by an average of 2292 steps (SD 3520.4; 95% CI 898-3370; *P*<.001), whereas the overweight-obese group did not show statistically significant changes (Table 2; Figure 1).

#### Engagement Measures

App usage frequency was significantly higher in the underweight-normal BMI group: over half of the participants in this BMI group used the *My measures* feature at least 50 times and used social features 150 times or more over the 6-month study duration (Table 2; Figure 1).

### Qualitative Findings

We conducted 32 individual interviews and 5 focus groups with 13 participants (20-45 min for each interview or focus group). Themes and subthemes did not differ between interviews and focus groups and consisted of (1) social comparison and networking (subthemes: social comparison, digital people watching and data sharing, social interactions, and negative aspects of social interactions), (2) self-monitoring (subthemes: self-monitoring of weight and BMI, self-monitoring of steps, and other fitness-related measures), and (3) Personalization and gamification. Social comparison and self-monitoring were seen as very distinct features by users, each with its positive and negative aspects, which in turn influenced motivation to use the app and engagement with the intervention. Personalization and gamification were commonly mentioned as desirable features to promote long-term engagement.

#### Social Comparison and Networking

##### Social Comparison

Most participants mentioned that social comparison of weight, BMI, or step count can be pointless if participants’ characteristics, goals, and lifestyle are not known (Textbox 1, quotes 1-3). However, most participants mentioned being motivated by the competition aspects enabled by social comparison, especially with regard to physical activity (Textbox 1, quote 4). The preferred type of comparison varied between individuals. Several participants in the underweight-normal category mentioned a preference for upward comparison in terms of fitness level, where they enjoyed comparing themselves with more active people in the lower BMI ranges (Textbox 1, quotes 5 and 6), whereas some participants in the overweight and obese categories indicated an inclination for comparison with individuals in similar or higher BMI categories to avoid feeling demotivated (Textbox 1, quotes 7-9).

**Illustrative quotes for social aspects related to weight management and physical activity.**

Social comparison:

- Common opinions
  - 1. Comparison never helped me at all. It never motivated me to add them to my friend list or anything, or compare my data with them regularly. Because I don't know them first of all, I don't know what their goal is, I don't know what their existing lifestyle is like or anything of that sort. I didn't know how to compare myself with them. (Male, 27, normal BMI)
  - 2. Feeling (...) disconnected from the numbers. (...) The numbers meant nothing really. [About the comparison of step counts and BMI with other participants] (Male, 20, high BMI)
  - 3. If everyone has the same goal (...) then it's beneficial, then the competitive aspect comes into play. (Male, 20, normal BMI)
  - 4. There were a lot of challenges that my friends also kept adding, like in [Fitbit] (...) with their friends and with my friends combined. So, it became like (...) 25, 30 people in one challenge. (...) Yeah, it was pretty good actually because it kept me motivated (...) the competition thing. (Male, 27, normal BMI)

- Upward comparison
  - 5. I prefer to just (...) compare myself with the more athletic people because I see myself as more of an athlete than not. (Male, 20, normal BMI)
  - 6. Somebody who you want to look up to and how they're using the device to get their goals—that will be a good value-add in my life. (Male, 27, normal BMI)

- Downward comparison
  - 7. I was looking at people who are similar and started off in a similar position, and then I just tried to keep track of who did - like how much walking they did and how their weight was going and so on. (Male, 22, high BMI)
  - 8. I reckon if you had groups, if you had all the fitness people together and then all of the average people together and then all the overweight people, it would feel more - you wouldn't feel as bad because you're finding out there are other people like you. Even if all of your group BMI is in the high range, you would - you'd feel like that there are other people like you who are trying. (Female, 20, high BMI)
  - 9. I went through [all the participants] and got all the ones with similar BMIs and that way at least my buddies were what I would consider similar, like (...) female and same BMI. (Female, 24, high BMI)

Digital *people watching* and data sharing

- 10. I like how we get to compare, and I like how other people don't get to see that I'm comparing against them. (...) So I don't look like a stalker. (Female, 20, normal BMI)
- 11. I guess there's not enough information in the app itself to kind of identify that person. I'm more than happy for other people to find out (...) how many steps I take and how much I weigh and how tall I am. Mainly because I don’t (...) have [a] close connection with them. If I did, I'd probably (...) be reserved in exposing some of that information. (Male, 30, high BMI)

Social interactions

- 12. For me it's the face-to-face and seeing someone and having to be accountable like that. (Female, 25, high BMI)
- 13. If you see that someone may be exercising around the same time as you, it might be you could turn it into a social thing where maybe you go to the gym or the park together. (Male, 22, high BMI)

Negative aspects of social interventions

- 14. When I realised I wasn't doing well against other people (...) I [wouldn’t] check it as much. (Female, 24, high BMI)
- 15. it can be a little bit demotivating when other people are not (...) taking this seriously (Female, 24, normal BMI)

##### Digital People Watching and Data Sharing

Several people mentioned that they enjoyed looking at other peoples’ measures without them knowing, a digital form of *people watching* enabled by the social network component of the app (Textbox 1, quote 10). Interestingly, sharing their own data, particularly weight and BMI, was disliked by some participants in the higher BMI categories, who mentioned a preference to remain anonymous in the network (Textbox 1, quote 11).

##### Social Interactions

Most participants highlighted the need to have real-world social connections for social support and accountability during the intervention. The desire for face-to-face support was particularly mentioned by people in the higher BMI categories, who indicated a need to have people to exercise with or a personal trainer to hold them accountable (Textbox 1, quotes 12 and 13). Participants in the normal BMI category frequently mentioned that they would have liked to have been able to invite their friends and family to the intervention, with some of them having even bought Fitbit trackers for family members to encourage their physical activity efforts (Textbox 1, quote 4).

##### Negative Aspects of Social Interventions

Two main negative aspects were mentioned with regard to social interventions. First, social comparison was seen by many participants as potentially leading to demotivation and negative emotions such as frustration, especially in the case of upward comparison in individuals with higher BMI, that is, comparison with a higher standard (lower BMI or more active individuals; Textbox 1, quote 14) [41]. Second, even highly motivated individuals could be negatively impacted by the lack of motivation or engagement from other individuals, such as participants not showing steps or weight data for periods of time (Textbox 1, quote 15). Both of these social causes for demotivation and negative emotions (upward comparison and lack of participation from other individuals) were mentioned by participants as leading to their lower engagement with the intervention.

#### Self-Monitoring

##### Self-Monitoring of Weight and BMI

Self-monitoring of weight and BMI was seen as *pointless* by several people in the normal weight range, although a few individuals mentioned liking the increased awareness of variations in their weight, especially when trying to gain muscle mass (Textbox 2, quotes 1 and 2). Some individuals in the higher BMI group saw the importance of weight monitoring and the benefits of using a wireless scale. However, individuals with both normal and higher BMI indicated a potential for negative emotions associated with self-monitoring, such as during periods of weight gain (Textbox 2, quotes 3 and 4).

**Illustrative quotes for self-monitoring of weight, BMI, and physical activity.**

Self-monitoring of weight and BMI:

- 1. the weight (...) doesn’t really matter to me that much. (Female, 24, normal BMI)
- 2. I think when I started, I was 58.8 and now I’m 59.8. (...) I didn’t want to lose weight (...). I wanted to gain muscle, so hopefully that one kilo is muscle. (Female, 20, normal BMI)
- 3. I didn't like the weight [monitoring]. (...) I didn't like looking at it there every single day, but I would check it. (Female, 31, high BMI)
- 4. When I was weighing myself (...) [and I felt] like I was gaining weight (...) I felt a bit stressed. (...) I was aware that I was gaining weight and then at times I was trying to eat less but then I ended up eating more chocolate, as in binge eating. (Female, 20, normal BMI)

Self-monitoring of steps and other fitness-related measures:

- 5. I would just open the app just to check (...) how many steps I [had] done. Because my step goal was about 10,000 steps and I would do that almost every day. (Male, 27, normal BMI)
- 6. At first, I was motivated and I was going for exercises because of the Fitbit just to achieve the goals. Then afterwards I lost interest. (Female, 20, high BMI)
- 7. Definitely at the beginning I was doing more, but then - I don't know - halfway through I just [got] bored of it. I needed something else. (...) I mean, life's busy, so you just forget about it. (Female, 22, high BMI)
- 8. In the beginning, yeah, I found it really cool. I could track my steps. I tried to complete my goal every day. (...) I was really, really motivated, I was tracking everything. But then I got bored of it really quickly and that's how I started to lose interest. (Female, 20, normal BMI)
- 9. I didn't like to be reminded if I didn't achieve a goal. I feel like there's so much in our lives that we [have to] do that (...) getting a reminder that you haven't achieved whatever your goal [was] I found that I didn't like that at all, to be honest. I found that to be demotivating. I just didn't want to know about it. (Female, 31, high BMI)
- 10. When I got the highest [number of] steps, I was pretty impressed. I took a screenshot. I was bragging about it. (...) Because that wasn't to do with weight or BMI or whatever, it was more of an equal playing field, I feel. (...) (Female, 20, high BMI)
- 11. I play basketball, so I [want to] compare myself to my rivals. Someone who plays in a similar position and skillset as me. But if it's just number of steps, how much walking I did on the day, I don't really want to compare. (...) Step count is just walking. I don't find walking a competitive thing. (Male, 31, high BMI)
- 12. [About the importance of measuring heart rate] Because with all the exercise, it changes up a lot - I wanted to see what my resting heart rate was.

##### Self-Monitoring of Steps and Other Fitness-Related Measures

Self-monitoring of steps was particularly useful for individuals who had a specific daily step count goal, independent of BMI group (Textbox 2, quote 5). Many participants mentioned that having a goal was a necessary but not sufficient condition for engagement. Specifically, while it was motivating in the beginning to try to achieve new goals, the novelty effect often wore off, with participants going back to previous patterns of physical activity (Textbox 2, quotes 6-8). During periods of decreased physical activity and lower step counts, several individuals mentioned staying away from checking the mobile app to avoid negative emotions and feelings of guilt (Textbox 2, quote 9).

Some participants in the higher BMI groups seemed to prefer self-monitoring of steps to self-monitoring of weight or BMI, owing to an increased sense of control over changes and a higher ability to progress and achieve goals (Textbox 2, quote 10). Self-monitoring of steps was seen as meaningless by other participants involved in types of physical activity where step counts were either irrelevant or not a good reflection of the effort or performance in the activity (eg, swimming, basketball; Textbox 2, quote 11). In addition, for a few individuals, wearing the Fitbit tracker was not possible while performing their sport (eg, rugby), decreasing their interest in the self-monitoring of steps. Users interested in increasing their fitness levels or muscle mass indicated a preference for other measurements in terms of self-monitoring (eg, heart rate, body fat percentage), instead of weight, BMI, or step counts (Textbox 2, quote 12).

#### Personalization and Gamification

Most participants mentioned similar preferences and desires for features, namely personalization and gamification. In particular, most people indicated the desire to have a higher degree of personalization in the app, in terms of the features, content, and feedback provided (Textbox 3, quotes 1-4). In addition, several participants highlighted the powerful role of gamification in increasing and sustaining motivation and engagement with the intervention (Textbox 3, quotes 5 and 6). Gamified features were considered important in both individual aspects related to weight management and physical activity (eg, badges for goal achievements) and in social aspects such as social comparison or competition (eg, leaderboards).

**Illustrative quotes for preferred and desired features—personalization and gamification.**

Preferred and desired features:

- Personalization
  - 1. You think about gym training sessions, you can have private sessions, you can have small group sessions or you can have a class session and you choose which one is best for you. The same [should happen] with the app. (Female, 20, normal BMI)
  - 2. I personally thought [the app] would give me recommendations on easier [exercises]. (...) Tailor it to me [according to the reaction to previous exercises] (Female, 20, high BMI)
  - 3. [Having health information] would be good, but it has to be personalized or customized to me, (...) my body type, (...) not like a general advice like [what is] BMI etc. (...) A lot of people can read about general information; but if it's personalized to you or customized to your needs, it's going to be more interesting and more reliable (...) (Male, 24, normal BMI)
- Gamification
  - 4. Whether to have one or multiple buddies, the choice depends on what works for the person. Maybe you can personalize it in some way. Maybe you can elect [to have] only one partner, or (...) to be put in a group. (Male, 20, normal BMI)
  - 5. You earn badges, which are just like token little things, and for some reason they just hook me in. (Female, 25, higher BMI)
  - 6. It loads your progress with a bar around the circle, then when it gets full it flashes. Like it's a celebration. (Female, 23, normal BMI)

## Discussion

### Principal Findings

In this mixed methods study, we found that participants in the overweight-obese group significantly lowered their BMI by almost 1 kg/m^2^ (3%) during the first month of the intervention, showing no statistically significant difference at the end of the intervention. Participants who were overweight-obese used the app significantly less compared with individuals in the underweight-normal BMI group, mentioning negative feelings and demotivation arising from social comparison, particularly upward comparison (with fitter people). Participants in the underweight-normal BMI group were avid users of self-monitoring, feedback, and social features within the app and significantly increased their daily step count over the 6-month study duration (by more than 2000 steps). Most participants mentioned the desire for a more personalized intervention.

### Social Comparison and Networking

In our study, social comparison and competition were preferred by participants in the underweight-normal BMI group (particularly upward comparison). Participants in the overweight-obese group mentioned a sense of vulnerability in sharing their data (eg, weight) in a social network and highlighted negative feelings and demotivation from upward comparison, similar to other studies [42], as well as a preference for face-to-face connections for support and accountability. These findings suggest that this group may benefit from being in a network of people with similar characteristics and goals. Previous research on weight and physical activity interventions has revealed that people seem more willing to participate in an online social network with others having common aspects (eg, real-world connections, similar goals, or fitness levels) [43-48].

This study builds on previous literature showing that online social networks can facilitate behavior change [15]. However, we found that user preferences regarding social features seem to be mixed, which indicates that such features should be available but optional, allowing users to control what information is shared and with whom [43,49,50]. Some people reported that they enjoy sharing their fitness achievements to receive praise and social support, in which case broadcasting to larger social networks such as Facebook seems to be helpful [51]. On the contrary, others did not like to share or participate in a social network but enjoyed *lurking*, that is, passively looking at content in social media without actively engaging [52]. This *digital people watching* effect promulgated by social media seemed to be enjoyed by some participants in our study.

### Self-Monitoring

Regular self-monitoring of weight and BMI was an activity that most participants in our study were not particularly fond of. Participants in the overweight-obese group recognized the importance of weight monitoring but still preferred focusing on steps, due to an increased sense of perceived control over changes and the desire to avoid negative emotions. Other studies have reported on users’ concerns about the potential for negative emotional and motivational impact when discrepancies between reality and goals are revealed in health apps [43]. Despite these concerns, regular self-monitoring of weight has been associated with weight loss and weight maintenance in individuals who are overweight and obese and is considered a healthy weight control strategy in people with a normal weight, with little evidence of adverse effects [53-58]. Given the importance of self-monitoring in behavior change [10,12], new strategies should be explored to promote engagement, such as decreasing the frequency of weighing to a level that individuals are able to maintain.

### Personalization and Gamification

Personalization was a recurrent topic endorsed by participants in our interviews and has been emphasized in other studies of digital interventions as a critical aspect for weight management and physical activity [49,50,59]. Nowadays, technology allows for increasing levels of personalization, having the potential to enhance engagement with digital health interventions [60] by molding them to be more relevant to users based on their personal characteristics or their motivation to change lifestyle [43,61]. In our study, people within and between different BMI groups showed different preferences regarding core features such as self-monitoring and social comparison, which, in turn, seemed to influence their engagement with the intervention. In addition, there was higher variability in the rate of BMI change across the overweight-obese group compared with the underweight-normal group over the duration of the intervention, suggesting that the overweight-obese group might benefit from higher degrees of personalization to accommodate the greater variability in this group. Currently, most commercial activity trackers and mobile apps offer one-size-fits-all interventions with minimal personalization, which may be a factor in their high drop-off rates [62,63].

### Strengths and Limitations

The strengths of this study include the objective measurement of outcomes using digital devices instead of self-reported data, the use of exploratory linear mixed effects analysis to better understand participants’ BMI changes weekly throughout the intervention period and complement pre-post data, and the use of postintervention interviews and focus groups to better understand pilot test results and assess the advantages and disadvantages of the intervention and its components. However, the findings should be interpreted in the context of some limitations. This was a quasi-experimental study with a single-arm pre-post design, and causation cannot be inferred from our results. Posthoc subgroup analyses and linear mixed effects analysis were exploratory and might be subject to type I error. As in other studies [15,18,64], engagement with the intervention decreased over the 6-month period, and there was a high degree of missing data over the duration of the study, which affected the quality of the mixed effects analysis. Baseline daily step count (average for the first week of the study before being able to access the study app) was considerably high in both BMI groups, which might reflect the novelty effect of starting to use an activity tracker. There was a predominance of men in the higher BMI categories at baseline, and a predominance of women in the lower BMI categories, which may explain the observed higher weight loss in men. We only used step count as a measure of physical activity, and other measures (eg, minutes of moderate-to-vigorous physical activity) might have shown different results. The results of our study should be interpreted in the context of concomitant use of the Fitbit app (which provided additional features such as goal setting, not available in the fit.healthy.me app) and the different goals participants may have had for joining the study (eg, increase physical activity, lose weight).

### Implications

Our study found different effects of the intervention in the underweight-normal BMI group and in the overweight-obese group, with participant perspectives also varying depending on the BMI group. The increase in step counts in the underweight-normal BMI group is promising given that any intensity of physical activity, including light intensity, is associated with a lower risk of premature mortality in a dose-response manner [5,65]. The short-term BMI decrease seen in the overweight-obese group may be due to the novelty factor of the intervention. Future research should explore whether it is possible to promote long-term physical activity and BMI changes in individuals who are overweight and obese with such an intervention and what types of interventions and features are associated with higher effectiveness (eg, interventions focusing on contextual and environmental factors, in addition to individual and social aspects; interventions offering personalization, such as the possibility to turn off social features or only allow for social comparison with similar individuals in terms of physical activity and BMI).

The importance of personalization was highlighted in this study by the heterogeneity of participant perspectives regarding intervention features. Smartphones and wireless trackers enable the collection of large volumes of personal data that can be leveraged to personalize interventions. Recent developments in artificial intelligence have led to the common use of machine learning models to optimize intervention content, timing, and delivery, based on users’ preferences, habits, and other individual and contextual data [66-68]. Future research should explore the impact of personalized features to better match individual preferences, barriers, and needs to promote higher engagement and enhance the effectiveness of interventions.

### Conclusions

A social networking mobile app connected to wireless tracking devices had different effects on participants in higher and lower BMI groups. Participants in the overweight-obese group showed a short-term decrease in their BMI that was not sustained after 1 month, and they used the app significantly less than participants in the underweight-normal BMI group, mentioning negative feelings with app use. Participants in the underweight-normal BMI group significantly increased their daily step count over the 6-month study duration. Most participants mentioned the desire for a more personalized intervention. Future research should explore the use of personalized features to better match individual preferences, barriers, and needs.

## Appendix

Multimedia Appendix 1 mHealth evidence reporting and assessment (mERA) guidelines and essential criteria.

Multimedia Appendix 2 Transparent reporting of evaluations with nonrandomized designs (TREND) statement checklist.

Multimedia Appendix 3 The consolidated criteria for reporting qualitative research (COREQ) checklist for reporting qualitative research.

Multimedia Appendix 4 Facebook post used as part of the recruitment strategy.

Multimedia Appendix 5 Screenshots of the mobile application “fit.healthy.me”.

Multimedia Appendix 6 Likelihood ratio tests to determine effect of predictors on BMI difference.

Multimedia Appendix 7 Linear mixed effects analysis of the weekly BMI change of each participant, using sex, weight baseline, and week as fixed effects.
