# Supplementary material for: A Mobile Social Networking App for Weight Management and Physical Activity Promotion: Results From an Experimental Mixed Methods Study
Source: J Med Internet Res. 2020 Dec 8;22(12):e19991. doi: 10.2196/19991 (PMC7755540; doi:10.2196/19991)
Supplement: Multimedia Appendix 2 [file jmir_v22i12e19991_app2.docx]

**Multimedia Appendix 2: TREND statement checklist**

Des Jarlais DC, Lyles C, Crepaz N. Improving the reporting quality of nonrandomized evaluations of behavioral and public health interventions: the TREND statement. *Am J Public Health.* 2004;94(3):361-366.

**
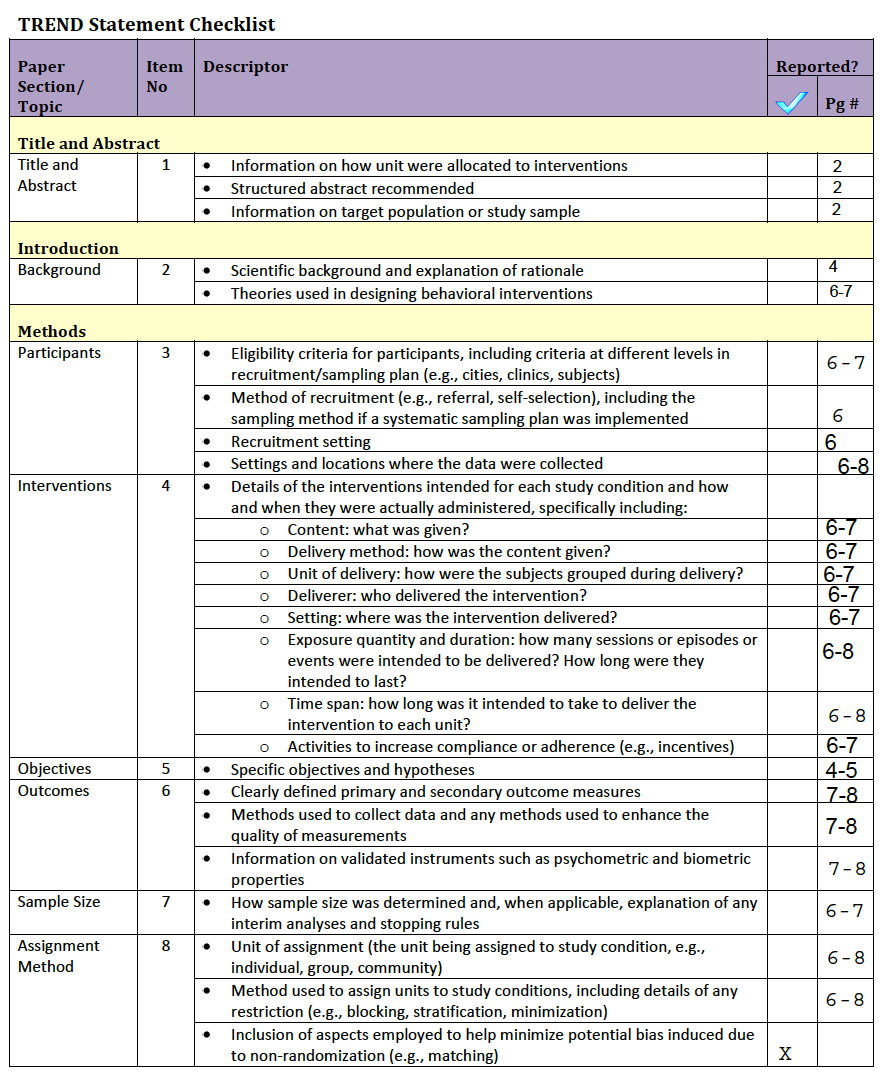
**

Methods> Study sample and recruitment

Methods> Study sample and recruitment;

Methods> Intervention

Methods> Quantitative data collection and analysis; Methods> Study sample and recruitment

**
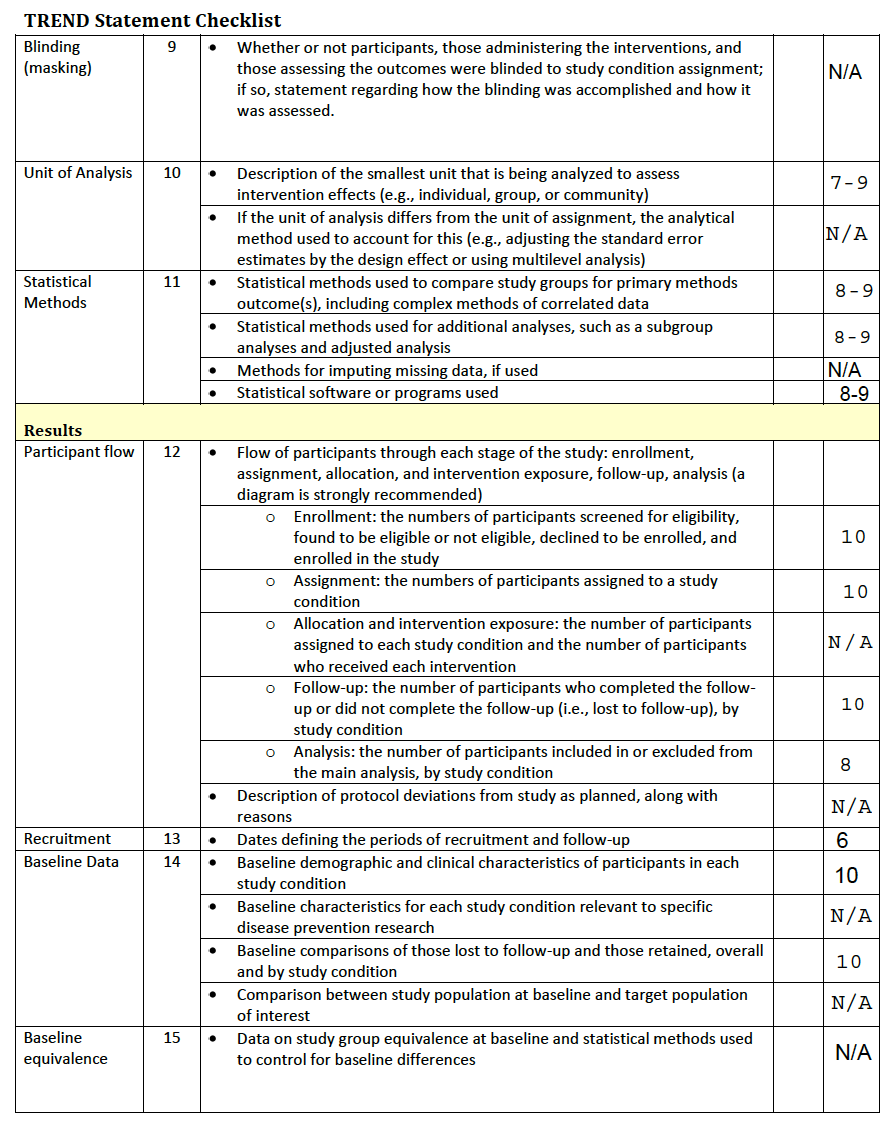
**

Methods> Quantitative data collection and analysis

Results> Study sample and

characteristics

Methods> Quantitative data collection and analysis

Methods> Study sample and recruitment

Results> Study sample and

characteristics

**
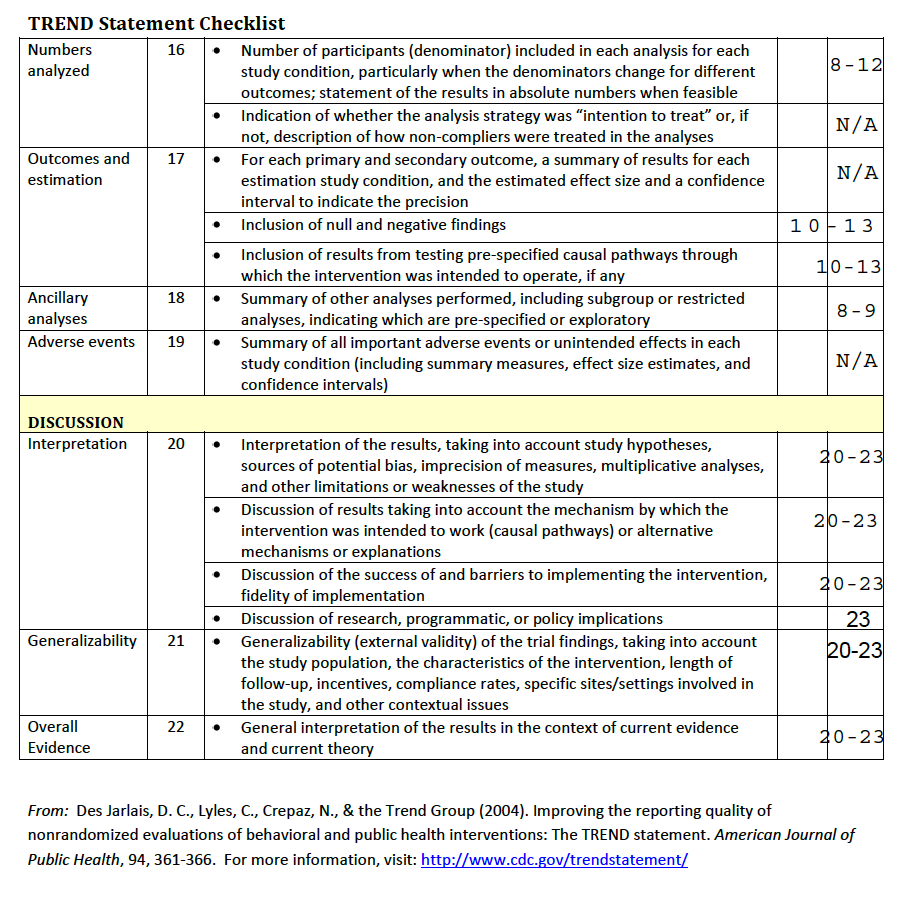
**

Methods>

Quantitative data collection and analysis

Results> Quantitative findings

Methods>

Quantitative data collection and analysis

Discussion
