# Supplementary figures and images for: A Mobile Social Networking App for Weight Management and Physical Activity Promotion: Results From an Experimental Mixed Methods Study
Source: J Med Internet Res. 2020 Dec 8;22(12):e19991. doi: 10.2196/19991 (PMC7755540; doi:10.2196/19991)

**Multimedia Appendix 4: Facebook post used as part of the recruitment strategy.**


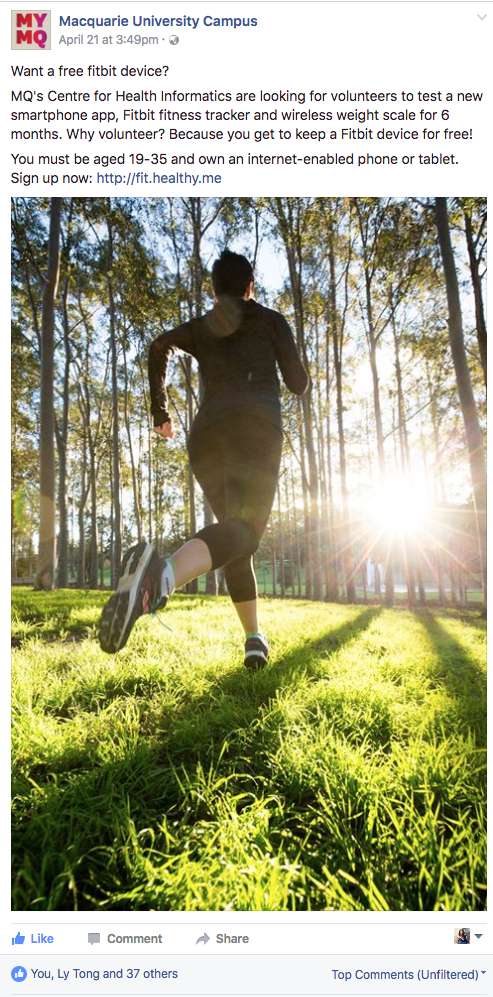

Supplement: Multimedia Appendix 4 [file jmir_v22i12e19991_app4.docx]

**Multimedia Appendix 5: Screenshots of the mobile application “fit.healthy.me”**

**
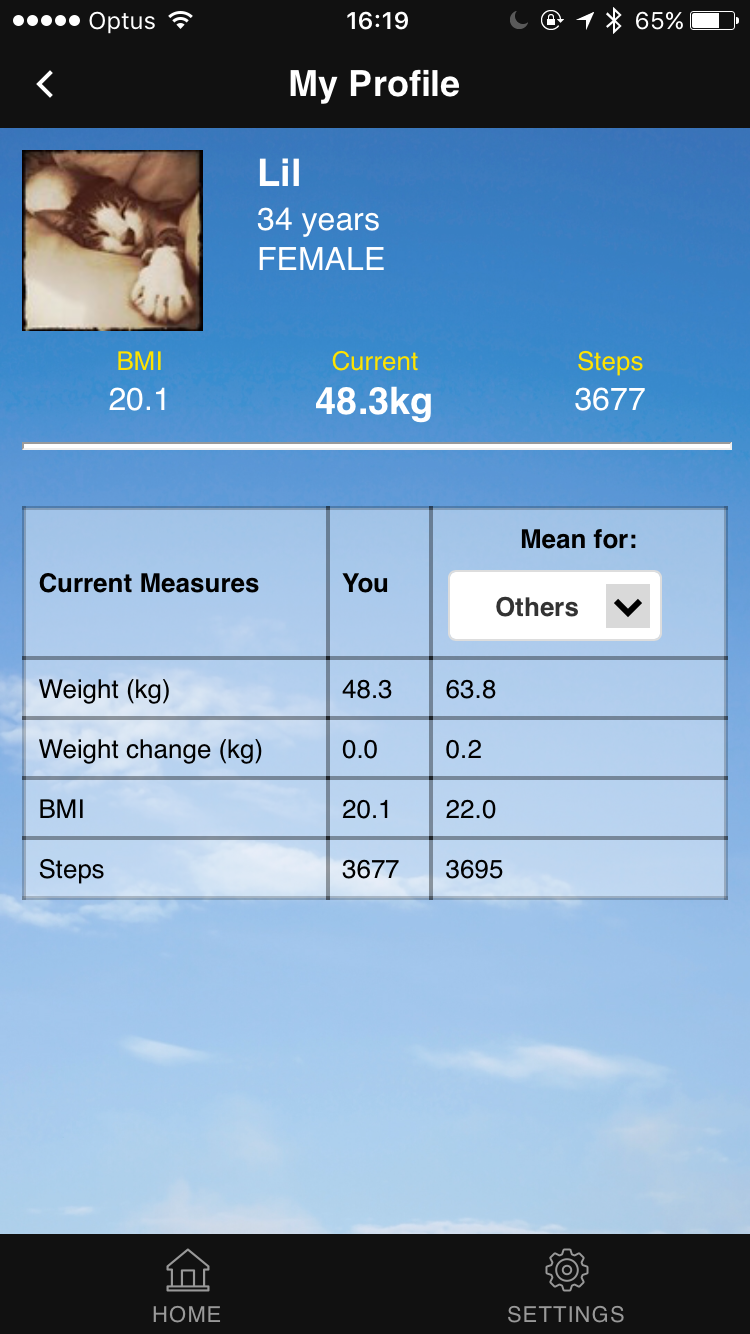

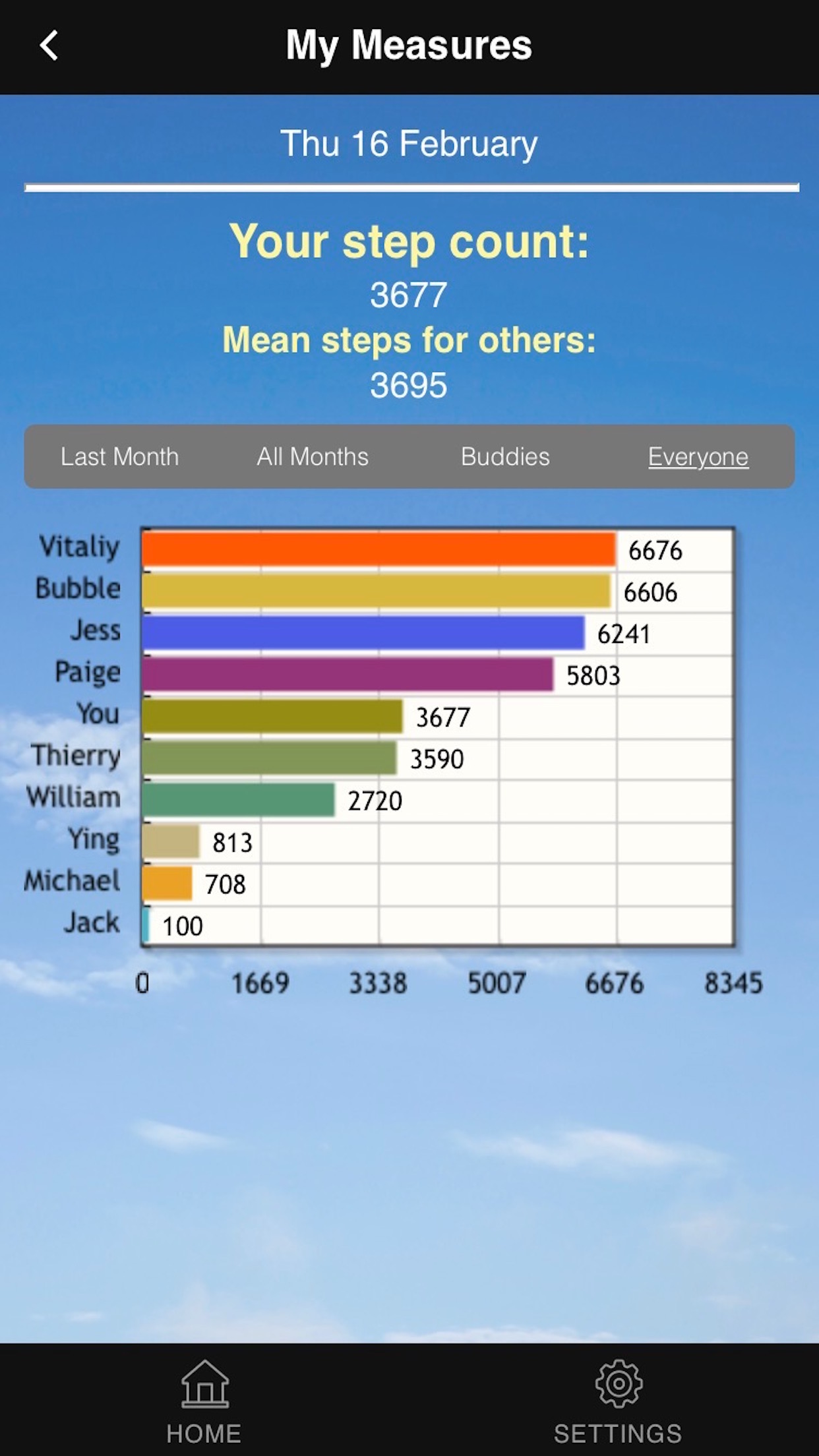
**

Supplement: Multimedia Appendix 5 [file jmir_v22i12e19991_app5.docx]
