# Supplementary material for: A Mobile Social Networking App for Weight Management and Physical Activity Promotion: Results From an Experimental Mixed Methods Study
Source: J Med Internet Res. 2020 Dec 8;22(12):e19991. doi: 10.2196/19991 (PMC7755540; doi:10.2196/19991)
Supplement: Multimedia Appendix 6 [file jmir_v22i12e19991_app6.docx]

**Multimedia Appendix 6: Likelihood ratio tests to determine effect of predictors on BMI difference.**

The baseline model was a random slopes model with BMI difference as the outcome, week as a fixed effect, intercepts for participants as a random effect, and by-subject random slopes for the effect of week. P-values for additional fixed effects variables were obtained by likelihood ratio tests of the baseline model against the baseline model with the effect in question (Table 1).

Baseline model:

$bmi diff \sim week+(1+week|participant)$

Example model with additional fixed effect:

$bmi diff \sim sex+ week+(1+week|participant)$

Sex ($\chi^{2}\left( 1 \right)=4.77, p=0.029$) and baseline weight ($\chi^{2}\left( 1 \right)=3.93, p=0.047$) had an effect on BMI difference for all participants, but not for the normal and the obese groups. Age, step difference, and scale usage did not have an effect on BMI difference (Table 1). We also tested various additional variables such as step difference between baseline and the final week, number of sessions of the app, various app usage sessions, but these were not found to improve the baseline model.

Table 1 - Likelihood ratio tests of the baseline model and the baseline model with the effect in question.

|  | **BMI**  **All Participants** | | **BMI**  **Underweight/Normal** | | **BMI**  **Overweight/Obese** | |
| --- | --- | --- | --- | --- | --- | --- |
|  | $\chi^{2}\left( 1 \right)$ | *P*-value | $\chi^{2}\left( 1 \right)$ | *P*-value | $\chi^{2}\left( 1 \right)$ | *P*-value |
| Sex | 4.77 | **0.029** | 1.09 | 0.297 | 2.19 | 0.139 |
| Baseline weight | 3.93 | **0.047** | 0.38 | 0.537 | 0.1 | 0.747 |
| Age | 0.03 | 0.864 | 0.16 | 0.686 | 0.71 | 0.401 |
| Step diff | 0.59 | 0.444 | 0.85 | 0.356 | 0.96 | 0.327 |
| Scale usage | 0.88 | 0.347 | 0.24 | 0.622 | 1.22 | 0.269 |
