# Supplementary material for: A Mobile Social Networking App for Weight Management and Physical Activity Promotion: Results From an Experimental Mixed Methods Study
Source: J Med Internet Res. 2020 Dec 8;22(12):e19991. doi: 10.2196/19991 (PMC7755540; doi:10.2196/19991)
Supplement: Multimedia Appendix 7 [file jmir_v22i12e19991_app7.docx]

**Multimedia Appendix 7: Linear mixed effects analysis of the weekly BMI change of each participant, using sex, weight baseline, and week as fixed effects.**

|  | **BMI**  **All participants** | | | | **BMI**  **Underweight/Normal** | | | | **BMI**  **Overweight/Obese** | | | |
| --- | --- | --- | --- | --- | --- | --- | --- | --- | --- | --- | --- | --- |
| **Predictors** | **Est** | **SE** | **CI** | ***P*** | **Est** | **SE** | **CI** | ***P*** | **Est** | **SE** | **CI** | ***P*** |
| **Intercept** | 0.11 | 0.32 | (-0.5,.735) | 0.72 | -0.51 | 1.14 | (-2.7,  1.7) | 0.66 | -0.7 | 0.72 | (-2.1,  0.7) | 0.32 |
| **Sex** | 0.26 | 0.15 | (0.05, 0.6) | 0.10 | 0.18 | 0.24 | (-0.3, 0.6) | 0.44 | 0.35 | 0.24 | (-0.1, 0.8) | 0.15 |
| **Baseline weight** | -0.01 | 0.00 | (-0.02, 0.01) | **0.14** | 0.00 | 0.01 | (-0.02, 0.03) | 0.76 | -0.00 | 0.01 | (-0.01, 0.01) | 0.80 |
| **Weekly BMI change** | -0.01 | 0.01 | (-0.02, 0.01) | **0.46** | 0.01 | 0.01 | (-0.00, 0.02) | 0.18 | -0.02 | 0.01 | (-0.05, 0.0) | 0.09 |
| **Random effects** | | | | | | | | | | | | |
| **Intercept variance** | 0.27 | | | | 0.16 | | | | 0.37 | | | |
| **Weekly BMI change variance** | 0.002 | | | | 0.001 | | | | 0.004 | | | |
| **Covariance**  **intercept – weekly BMI change** | 0.28 | | | | 0.05 | | | | 0.33 | | | |
